# Supplementary material for: Genome wide association study of clinical duration and age at onset of sporadic CJD
Source: PLoS One. 2024 Jul 26;19(7):e0304528. doi: 10.1371/journal.pone.0304528 (PMC11280162; doi:10.1371/journal.pone.0304528)
Supplement: S1 Text — (DOCX) [file pone.0304528.s001.docx]

**Patient recruitment and phenotypes**

Samples from patients with prion diseases were provided by specialist or national surveillance centres. A diagnosis of probable or definite sporadic CJD was required according to the contemporary widely accepted epidemiological criteria. These criteria have evolved over time, principally to include the recognition of the importance of MRI brain imaging, and the cerebrospinal fluid Real-Time Quaking Induced Conversion Assay (RT-QuIC) in diagnosis. As there was no restriction on the calendar date of diagnosis many patients were diagnosed using previous versions of diagnostic criteria with similarly high levels of specificity. Ethical approval for research studies was provided by London – Harrow Research Ethics Committee. Clinical duration was defined as the time from first symptom to death (months). Age at clinical onset was defined as the age at the time of the first symptom (years).
