# Supplementary figures and images for: Genome wide association study of clinical duration and age at onset of sporadic CJD
Source: PLoS One. 2024 Jul 26;19(7):e0304528. doi: 10.1371/journal.pone.0304528 (PMC11280162; doi:10.1371/journal.pone.0304528)

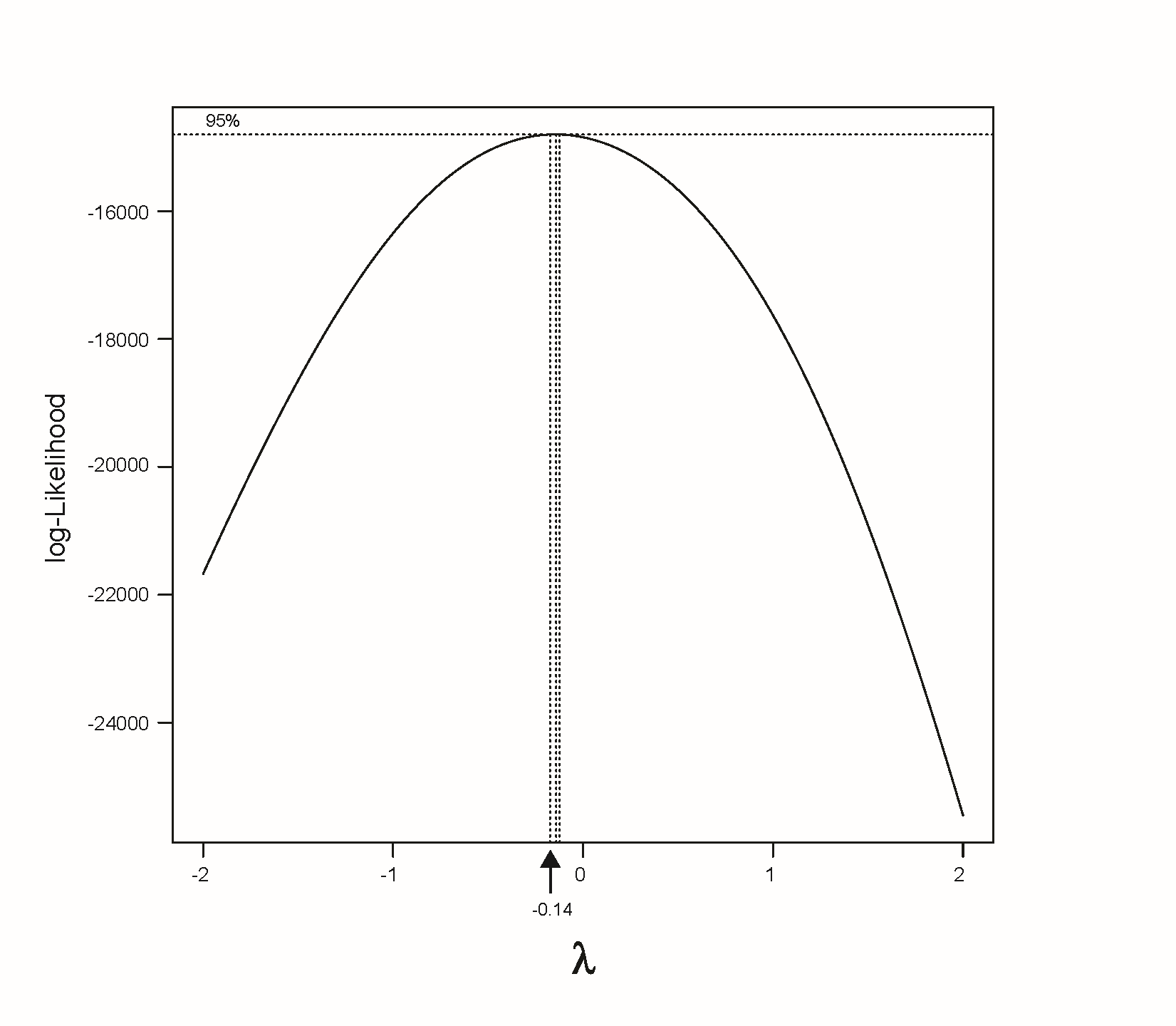

Supplement: S1 Fig — (TIF) [file pone.0304528.s002.tif]

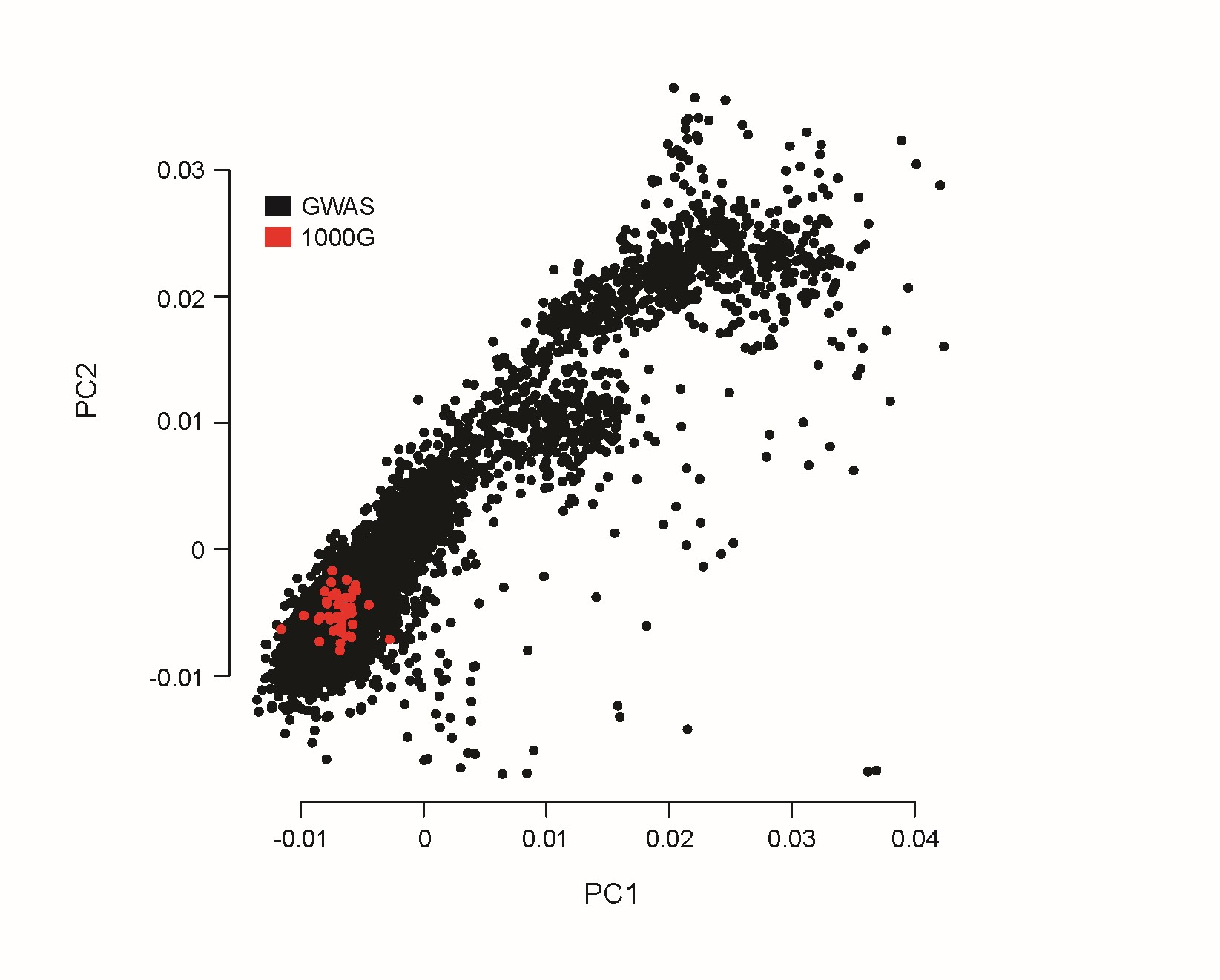

Supplement: S2 Fig — (TIF) [file pone.0304528.s003.tif]

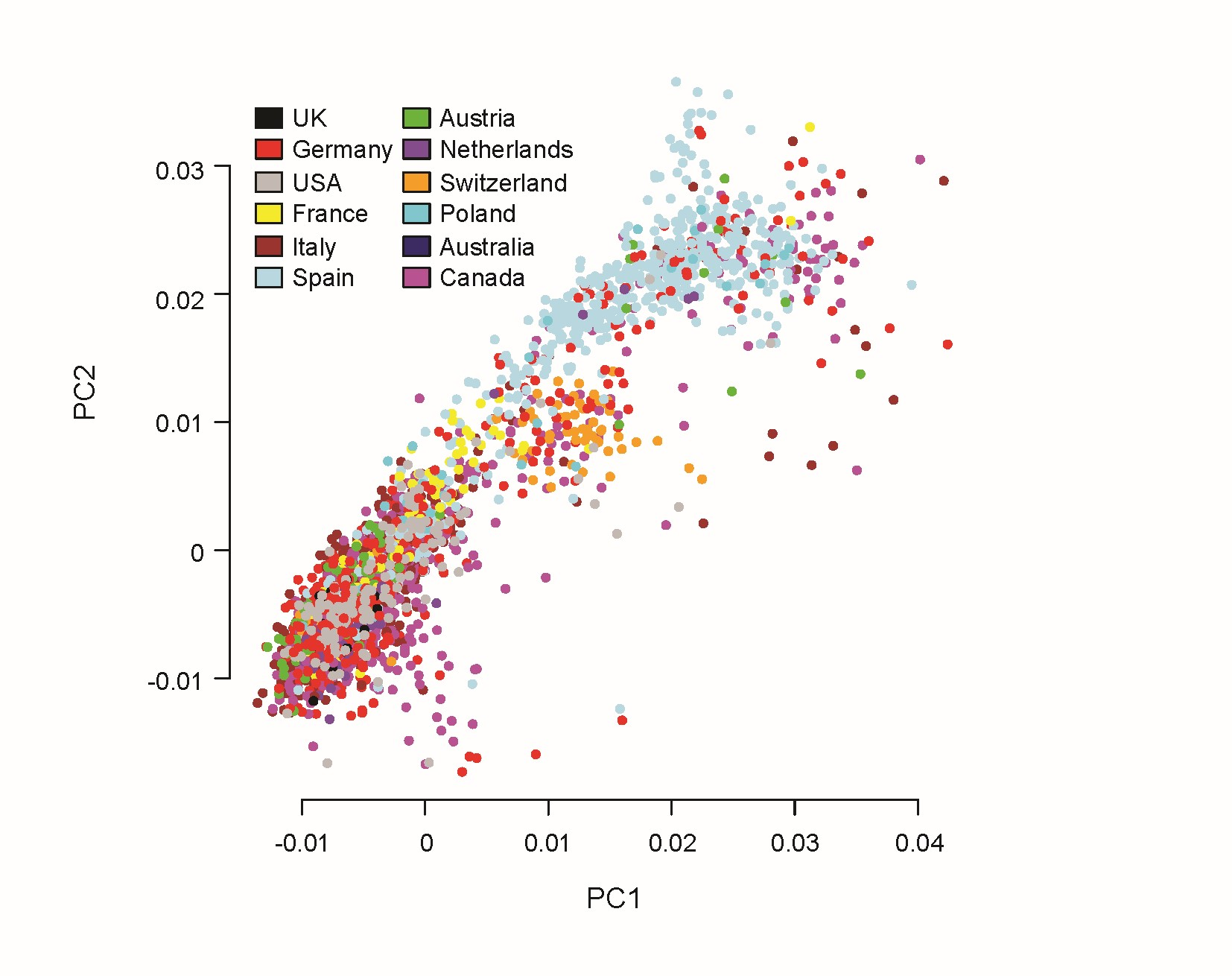

Supplement: S3 Fig — (TIF) [file pone.0304528.s004.tif]

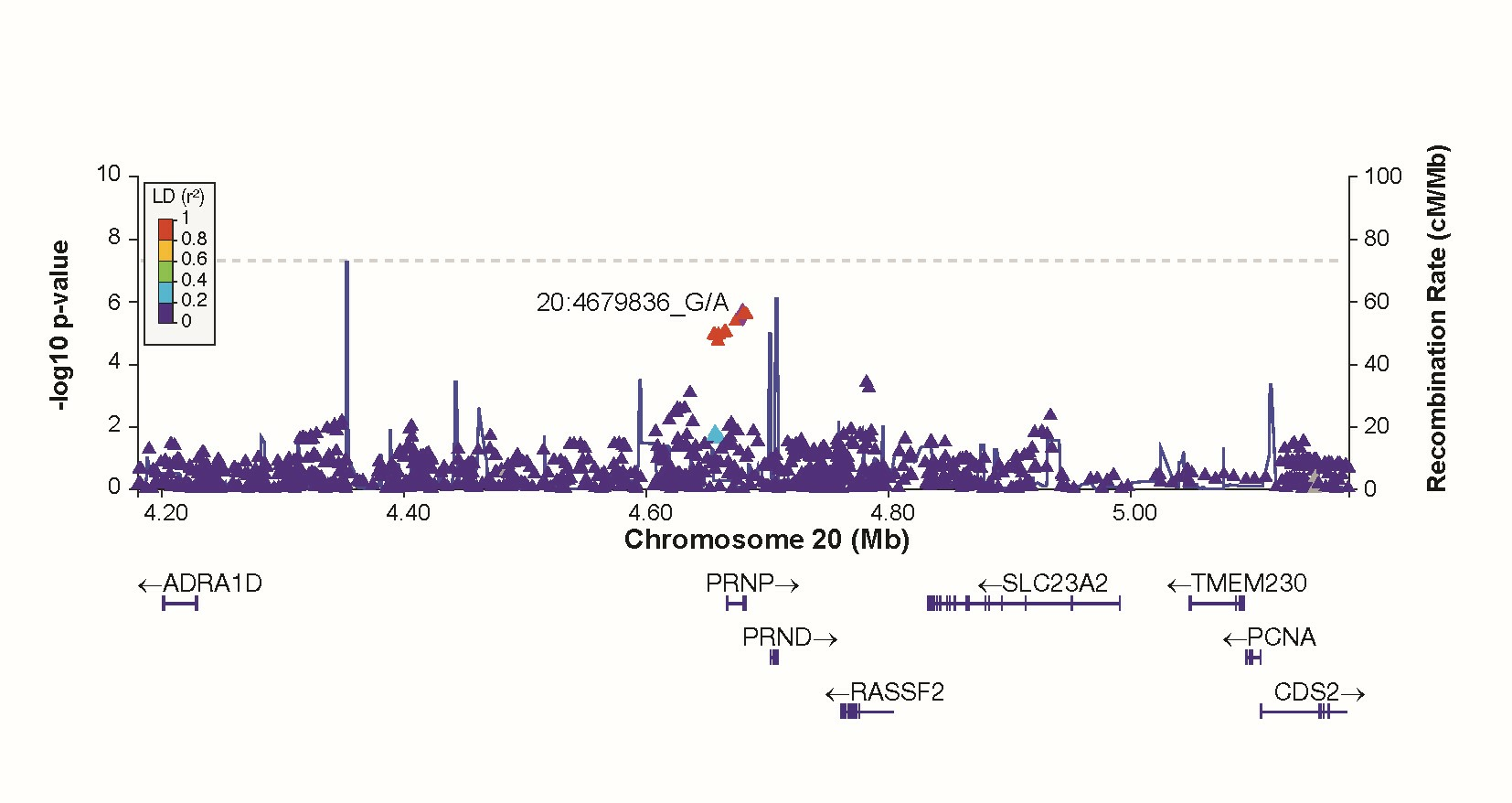

Supplement: S4 Fig — (TIF) [file pone.0304528.s005.tif]

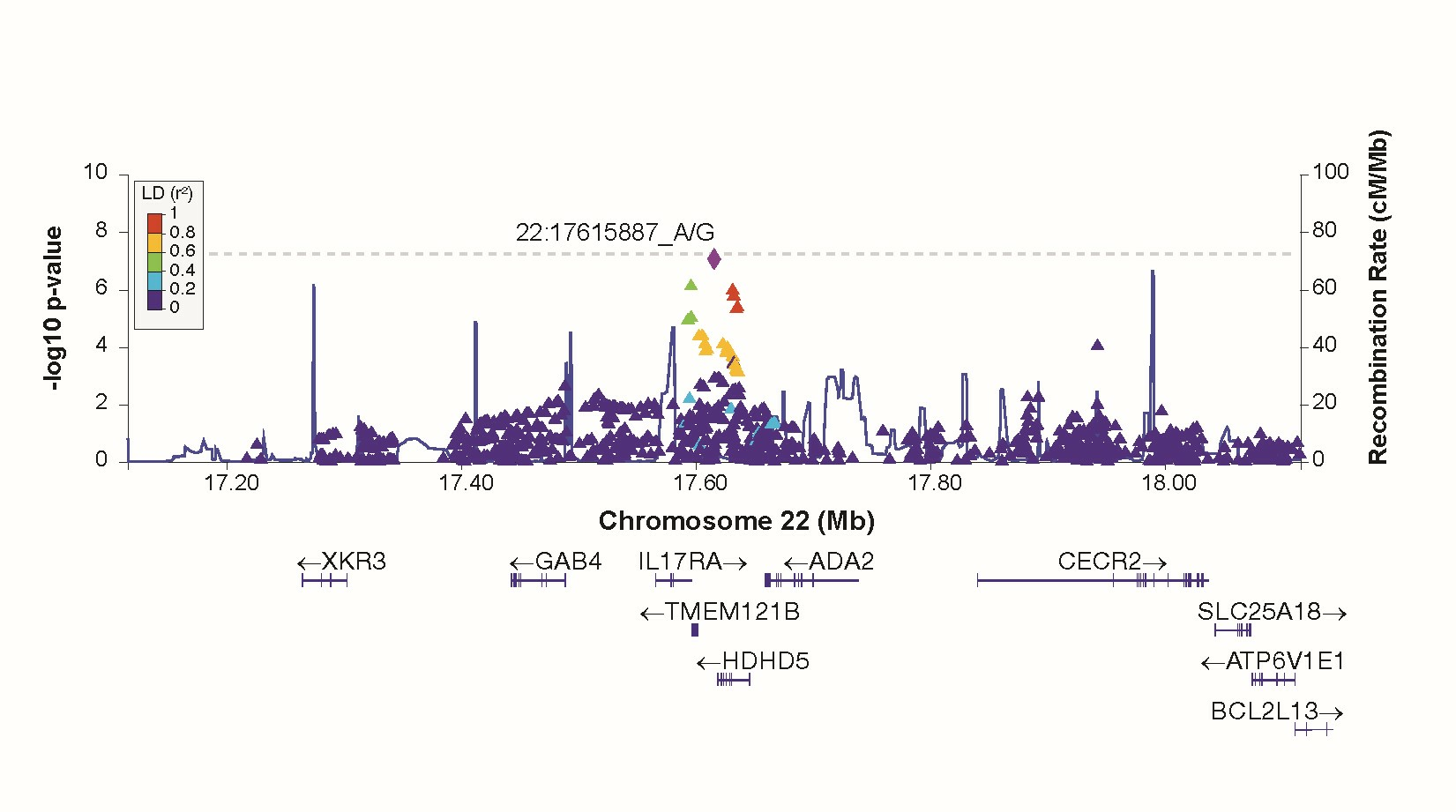

Supplement: S5 Fig — (TIF) [file pone.0304528.s006.tif]

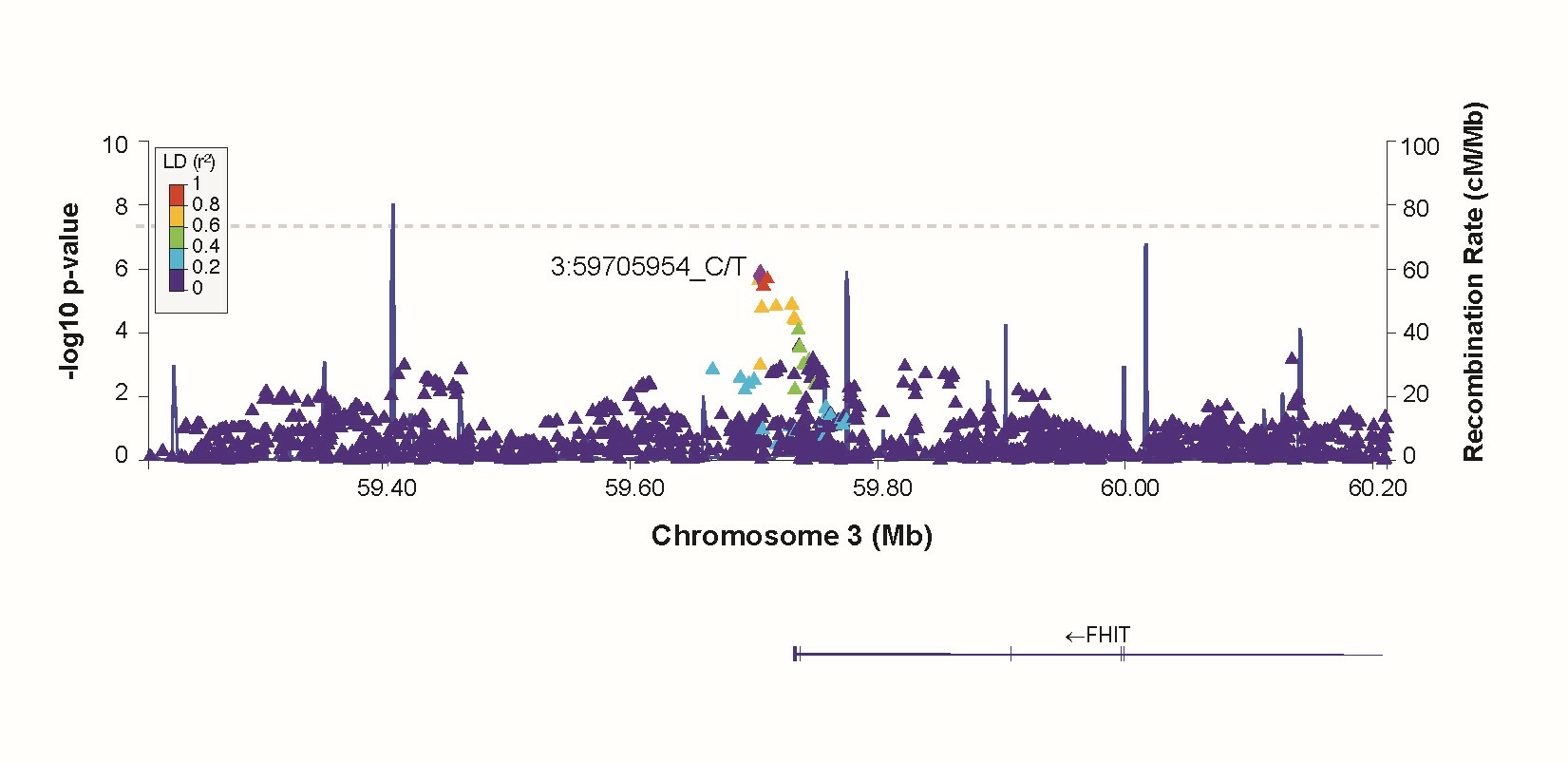

Supplement: S6 Fig — (TIF) [file pone.0304528.s007.tif]

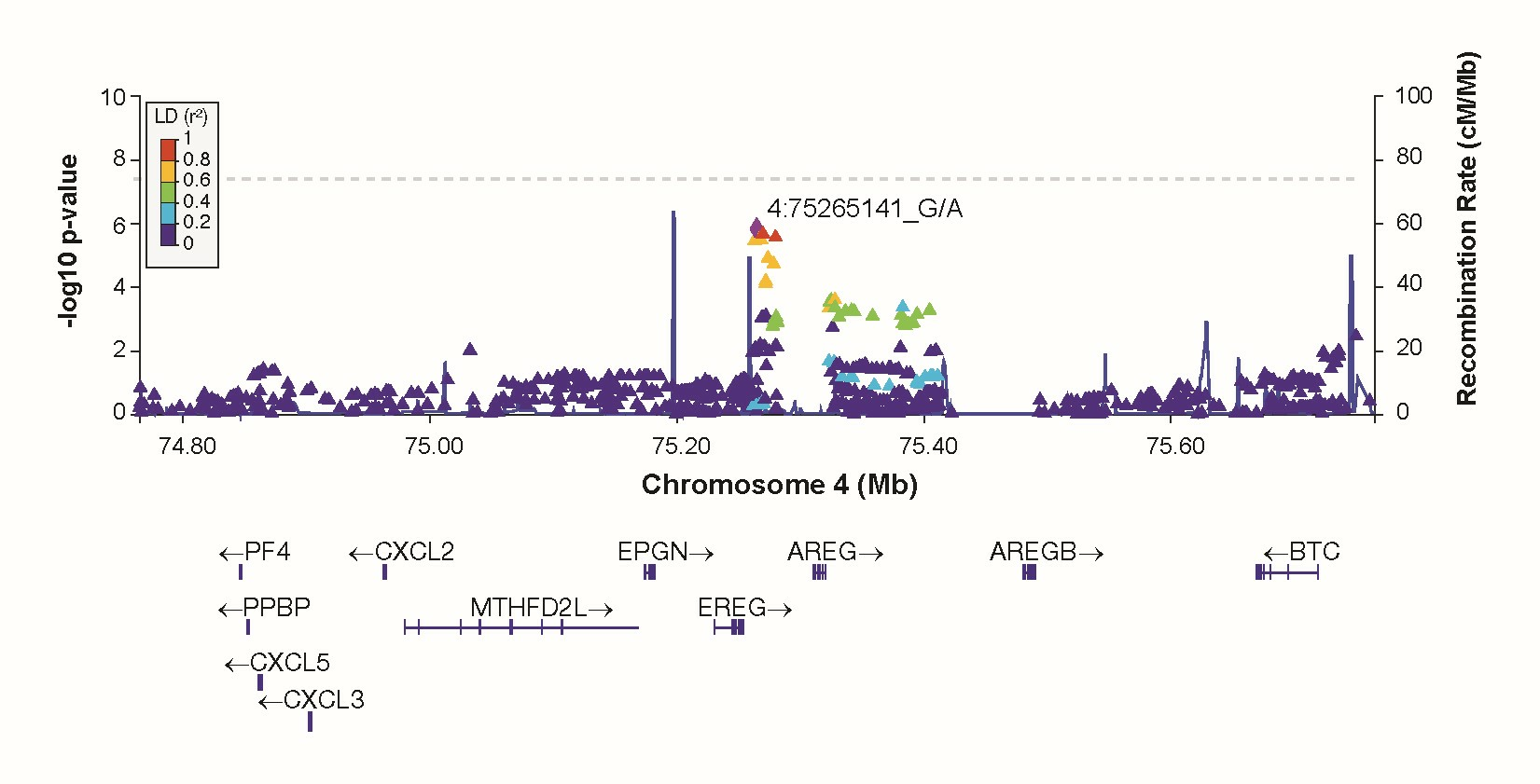

Supplement: S7 Fig — (TIF) [file pone.0304528.s008.tif]

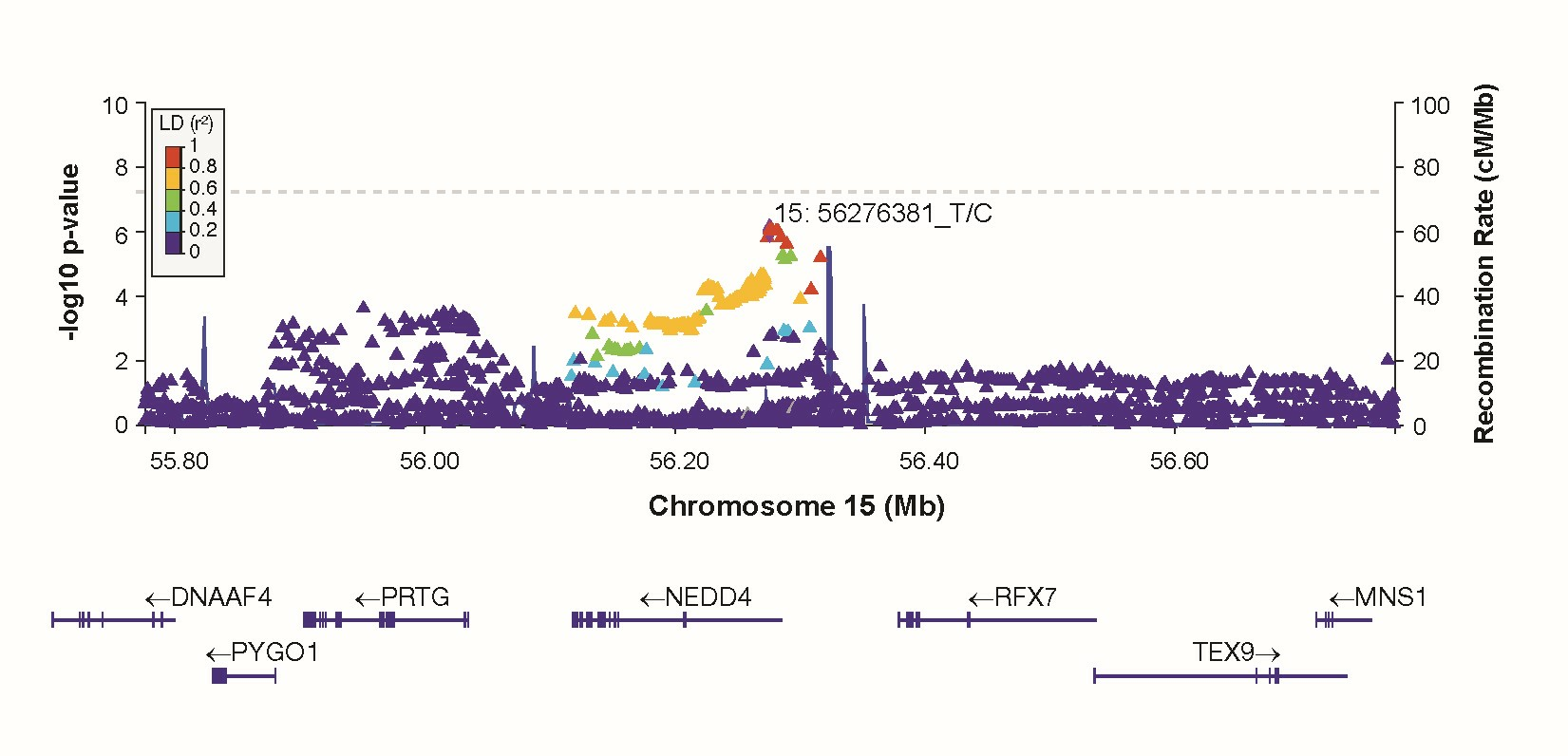

Supplement: S8 Fig — (TIF) [file pone.0304528.s009.tif]

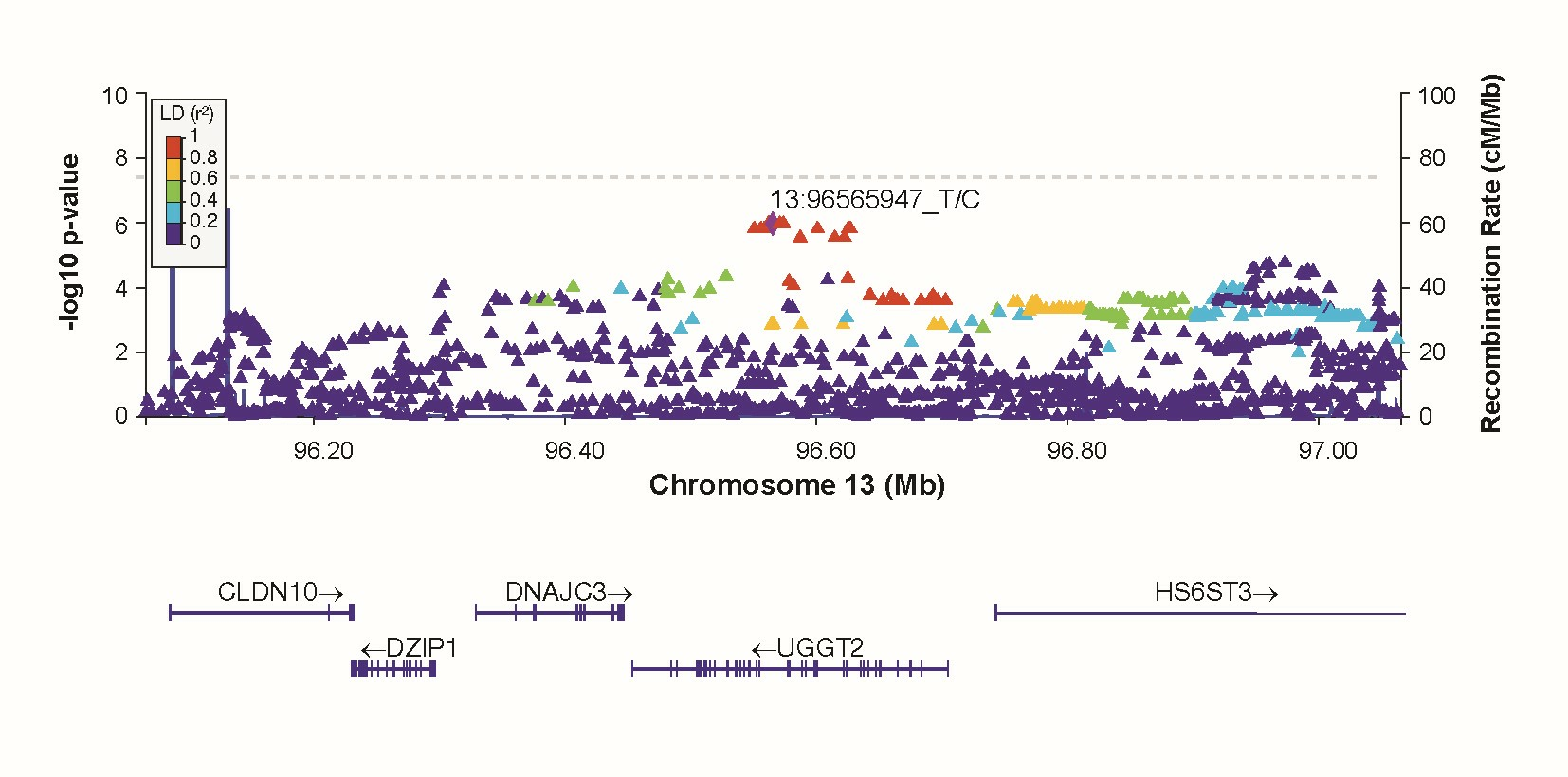

Supplement: S9 Fig — (TIF) [file pone.0304528.s010.tif]

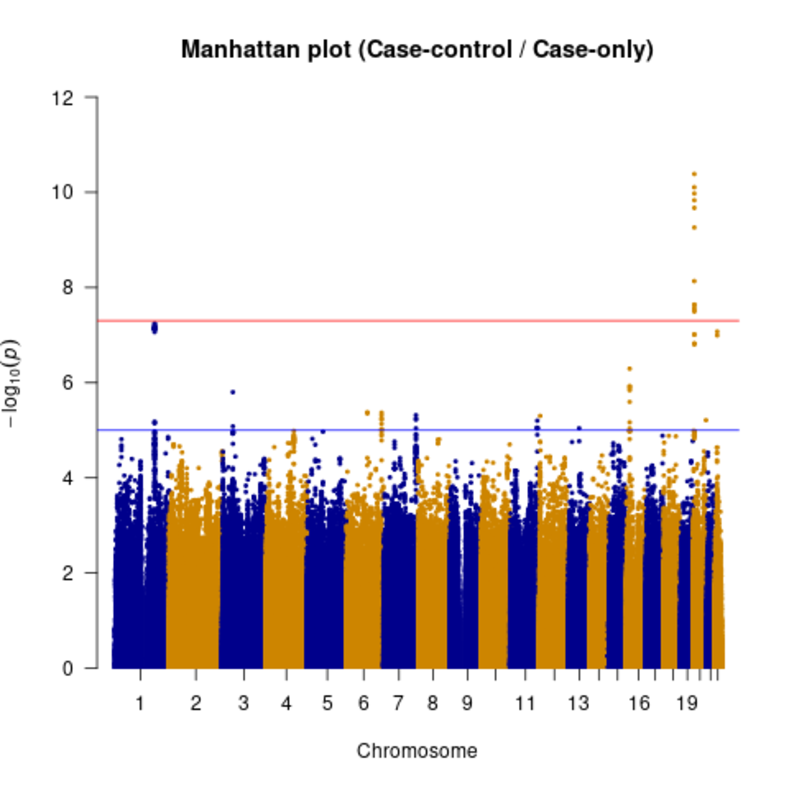

Supplement: S10 Fig — (TIF) [file pone.0304528.s011.tif]

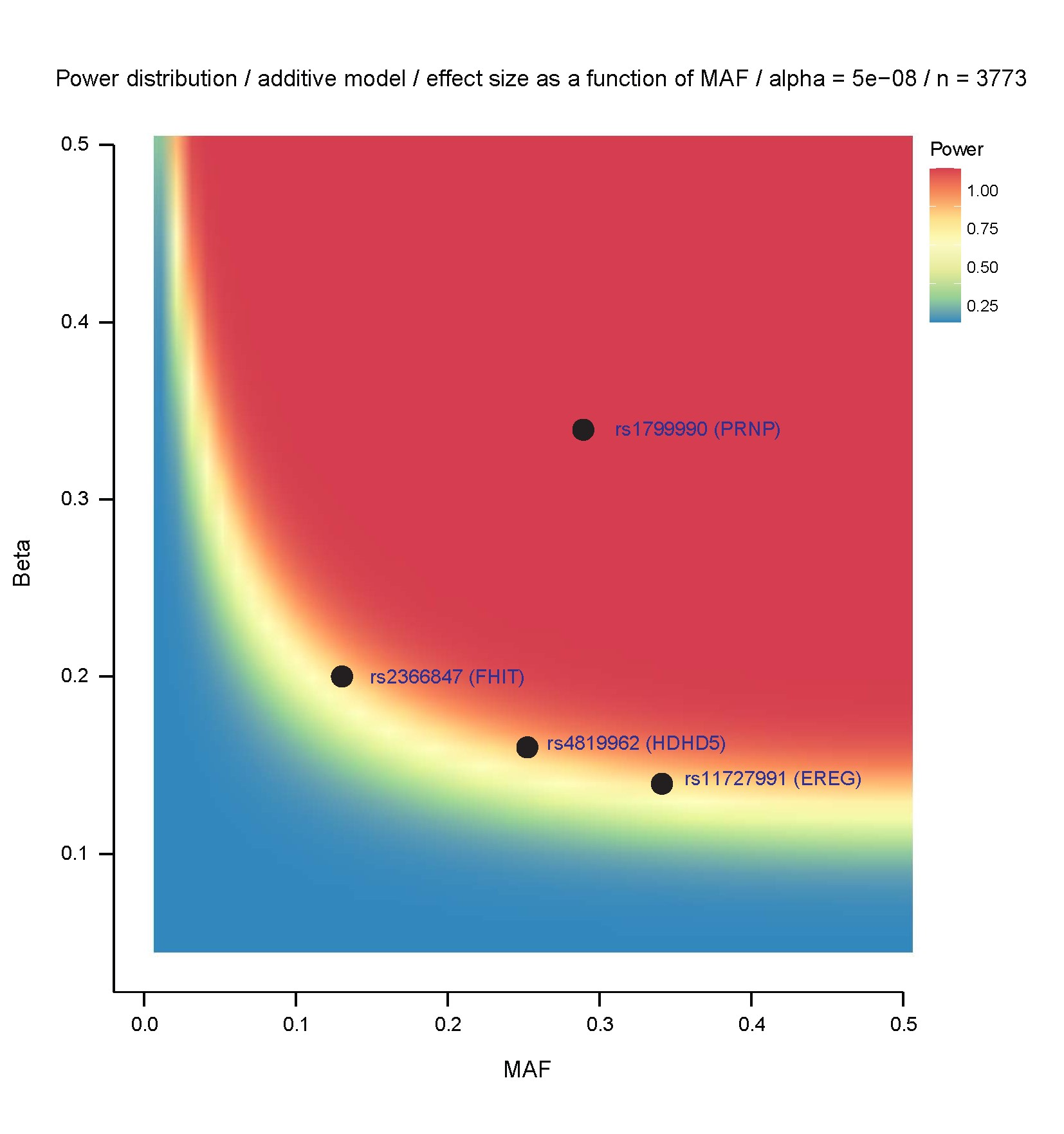

Supplement: S11 Fig — (TIF) [file pone.0304528.s012.tif]

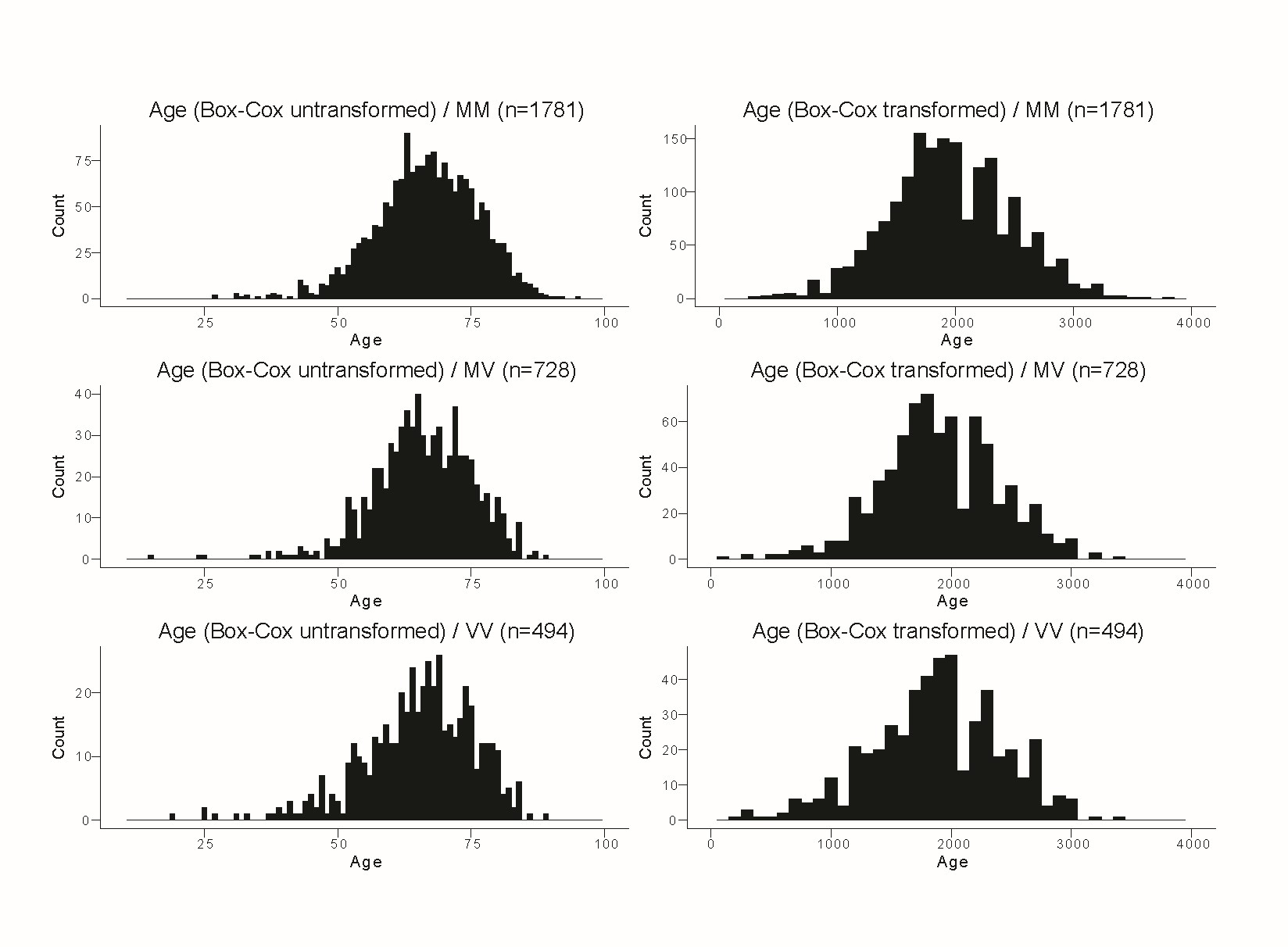

Supplement: S12 Fig — (TIF) [file pone.0304528.s013.tif]

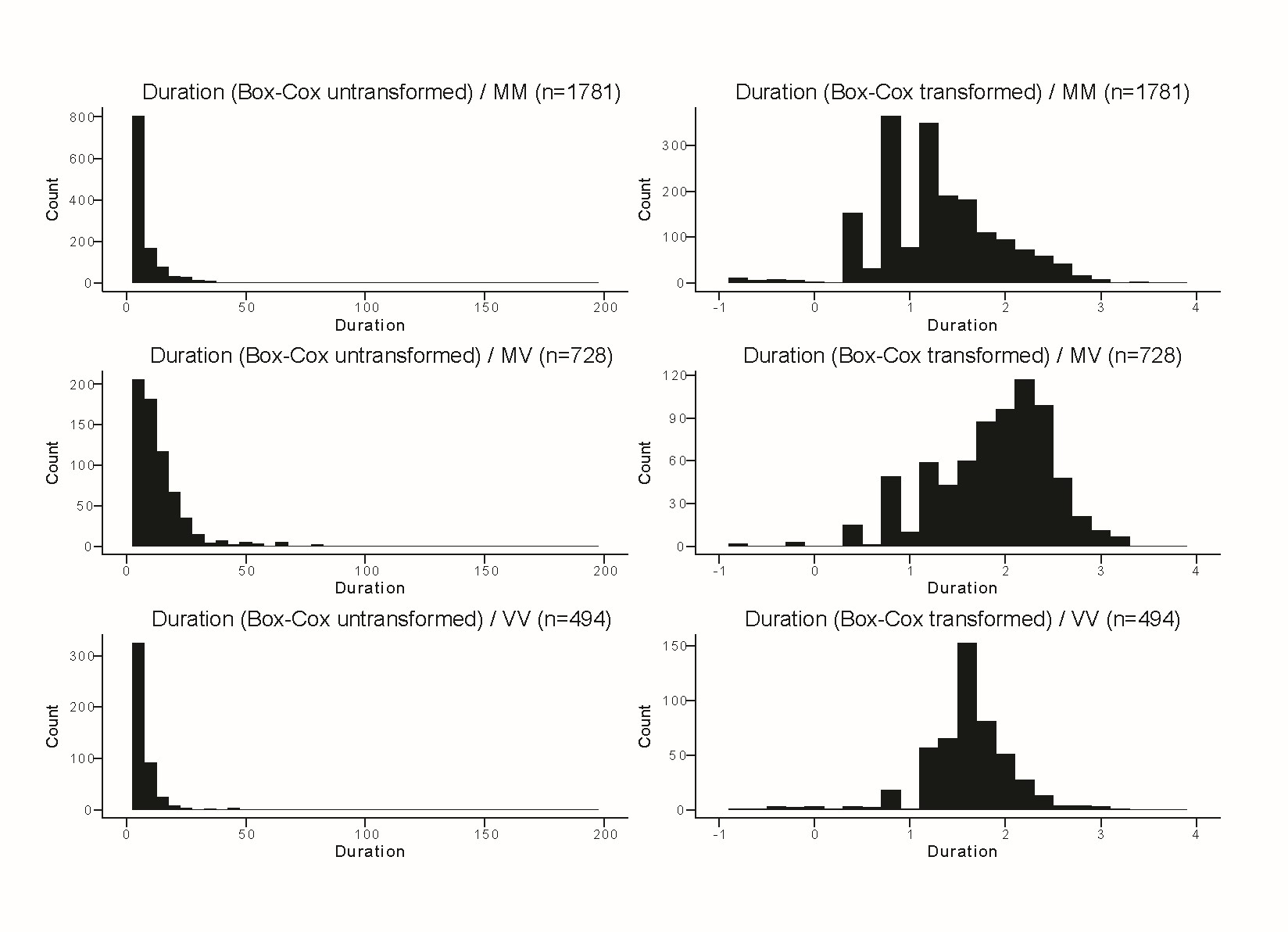

Supplement: S13 Fig — (TIF) [file pone.0304528.s014.tif]
