# Supplementary material for: Genome wide association study of clinical duration and age at onset of sporadic CJD
Source: PLoS One. 2024 Jul 26;19(7):e0304528. doi: 10.1371/journal.pone.0304528 (PMC11280162; doi:10.1371/journal.pone.0304528)
Supplement: Attachment — Submitted filename: Response to reviewers.docx [file pone.0304528.s024.docx]

We thank Reviewer #1 for his positive comments.

We thank Reviewer #2 for his positive comments.

In relation to his comment that a larger sample size is unlikely to result in the identification of potential modifiers, we would like to state that we identified SNPs very close to the genome wide significance level of 5*10^-8^ in gene *HDHD5.* We have and are currently collecting additional samples and we cannot rule out the possibility that the addition of these samples might confirm *HDHD5* as a risk locus for CJD in the future.

The second comment relates to the publication by Poleggi et al. 2018 identifying CYP4X1 as a candidate disease modifier for sCJD. We have addressed this in the Discussion section of the manuscript.

We also added examples of other GWAS studies of other neurological diseases with age-at-onset phenotype to the Discussion section. We have cited two publications, one by Li et al (2023) (Alzheimer’s disease) and another one by Blauwendraat et.al (2019) (Parkinson’s disease).
